# Risk factor analysis and prediction of postoperative clinically relevant pancreatic fistula after distal pancreatectomy

**DOI:** 10.1186/s12893-023-01907-w

**Published:** 2023-01-11

**Authors:** Chenchen He, Yibing Zhang, Longfei Li, Mingda Zhao, Chunhui Wang, Yufu Tang

**Affiliations:** 1 Department of Hepatobiliary and Thyroid Surgery, General Hospital of Northern Theater Command, Shenyang, 110000 China; 2 China Medical University, Shenyang, 110122 China; 3 Department of Medical Affairs, The General Hospital of Northern Theater Command, Shenyang, China

**Keywords:** Distal pancreatectomy, Pancreatic fistula, Risk factors, Nomogram

## Abstract

**Objective:**

Postoperative pancreatic fistula (POPF) following distal pancreatectomy (DP) is a serious complication. In the present study, we aimed to identify the risk factors associated with clinically relevant postoperative pancreatic fistula (CR-POPF) and establish a nomogram model for predicting CR-POPF after DP.

**Methods:**

In total, 115 patients who underwent DP at the General Hospital of Northern Theater Command between January 2005 and December 2020 were retrospectively studied. Univariate and multivariable logistic regression analyses were used to identify the independent risk factors associated with CR-POPF. Then, a nomogram was formulated based on the results of multivariable logistic regression analysis. The predictive performance was evaluated with receiver operating characteristic (ROC) curves. Decision curve and clinical impact curve analyses were used to validate the clinical application value of the model.

**Results:**

The incidence of CR-POPF was 33.0% (38/115) in the present study. Multivariate logistic regression analysis identified the following variables as independent risk factors for POPF: body mass index (BMI) (OR 4.658, P = 0.004), preoperative albumin level (OR 7.934, P = 0.001), pancreatic thickness (OR 1.256, P = 0.003) and pancreatic texture (OR 3.143, P = 0.021). We created a nomogram by incorporating the above mentioned risk factors. The nomogram model showed better predictive value, with a concordance index of 0.842, sensitivity of 0.710, and specificity of 0.870 when compared to each risk factor. Decision curve and clinical impact curve analyses also indicated that the nomogram conferred a high clinical net benefit.

**Conclusion:**

Our nomogram could accurately and objectively predict the risk of postoperative CR-POPF in individuals who underwent DP, which could help clinicians with early identification of patients who might develop CR-POPF and early development of a suitable fistula mitigation strategy and postoperative management.

## Introduction

Distal pancreatectomy (DP) is the standard procedure for the removal of benign or malignant tumours from the pancreatic body or tail. With the development of preoperative management and improvements in surgical techniques, the mortality associated with DP has decreased in the last decade, yet the major morbidity rate remains high, especially in patients who undergo open approaches [1–8]. The most common and severe complication is postoperative clinically relevant pancreatic fistula (CR-POPF), which further causes intraperitoneal abscesses and subsequent lethal haemorrhage [9]. Great efforts have been made to reduce the incidence of CR-POPF in the last decade [4, 10–15]. However, the incidence of CR-POPF after DP is still high, ranging from 9.7 to 39% [4, 10–18]. Therefore, how to effectively reduce the incidence of CR-POPF and conduct timely treatment thereafter is an urgent clinical issue to be solved.

Risk prediction models are increasingly advocated as tools to assist risk stratification and guide prevention and treatment decisions relating to common health conditions [19, 20]. Risk prediction models were constructed to predict the risk of POPF following pancreaticoduodenectomy (PD). For example, the fistula risk score (FRS) and alternative fistula risk score (a-FRS) have been widely reported to predict CR-POPF with high accuracy [21–25]. The use of FRS to determine the risk of CR-POPF can facilitate management-related decision-making, especially drainage strategy [26–29]. The nomogram was also developed to predict CR-POPF following PD [30–33]. It should be noted that the incidence of CR-POPF is higher in patients who undergo DP than in those who undergo PD, but risk prediction models are rarely constructed for CR-POPF in patients who undergo DP. Although numerous risk factors have been previously associated with CR-POPF following DP, a single risk factor does not accurately predict CR-POPF. The development of a risk prediction for CR-POPF following DP is of utmost importance and could help surgeons anticipate, identify, and manage this severe complication from the outset.

In the present study, we aimed to analyse the risk factors contributing to CR-POPF following DP and then develop and validate a nomogram for predicting CR-POPF.

## Methods

### Patients

Between January 2005 and December 2020, data on consecutive patients who underwent DP were retrospectively collected from the electronic medical record system at the General Hospital of Northern Theater Command. Eligibility criteria were as follows: (1) DP procedure performed; (2) complete preoperative examinations and postoperative 90-day follow-up data; (3) and no history of pancreatectomy. This study was approved by the institutional Ethics Committee of the General Hospital of Northern Theater Command (No.: Y (2021) 056). Written informed consent was obtained from all the patients or patients’ relatives before the surgery.

### Surgical procedures and postoperative management

The surgical methods of an open approach and laparoscopic approach were included in the present study. The choice of surgical procedures was decided by consultation among surgeons of our department, and the underlying disease condition was evaluated by preoperative radiological imaging. All resections were performed by senior consultant surgeons with more experience (≥ 20 pancreatectomies per year). The operative techniques were conducted as reported in previous studies [34]. During the operation, a nasogastric tube (NGT) was placed. Splenectomy was performed when malignant neoplasms were diagnosed by preoperative evaluation or spleen-preserving surgery could not be performed because of invasion of blood vessels. Two tubes were generally placed at the end of an operation for drainage of fluid, one tube near the pancreatic stump remnant and another drainage tube in the surgical field.

All patients received routine anti-infection, inhibition of pancreatic exocrine secretion, inhibition of gastric acid secretion, and nutritional support after surgery. Routine blood and biochemical examinations were performed on postoperative day (POD) 1 and then every 3 days until discharge. The amylase level in drainage fluid was routinely measured on POD 3, 5 and 7. Abdominal computed tomography (CT) was usually performed on POD 5 and any time patients had complex abdominal complications. The drainage tube was removed according to the Chinese consensus [35].

### Clinicopathological variables

Based on previous studies and the potential association between variables and CR-POPF, clinicopathological variables were selected. All clinicopathological characteristics were extracted from electronic medical records. BMI was calculated as weight (in kg)/height^2^ (in m^2^). Pancreatic thickness was measured as previously reported [36]. In brief, pancreatic thickness was measured at the resection line in preoperative computed tomography (CT) by 1 researcher who was blinded to the POPF result. The resection line was evaluated with postoperative CT at 5 days after the operation. The pancreatic texture was determined by the surgeon’s tactile response and confirmed from the histopathological reports based on the fibrosis grade of the pancreatic tissues. When the patients underwent laparoscopic DP, the texture of the pancreas was determined by the tactile feedback of the instrument and was reassured after being pulled out from the abdominal cavity. CR-POPF was defined in accordance with the updated 2016 ISGPF consensus guidelines [9]. Briefly, an external fistula with a drain output of any measurable volume of fluid after postoperative Day 3 with an amylase level more than three times the upper limit was associated with a clinically relevant development/condition related directly to POPF. The clinicopathological variables in this study are reported in Table 1.

**Table 1:** Clinicopathological characteristics of 115 patients undergoing DP

Variable	All patients (n = 115)
Gender, n (%)	
Male	34 (29.6)
Female	81 (70.4)
Age (y), median (IQR)	53.0 (45.5–62.0)
BMI (kg/m^2^), n (%)	
< 25	63 (54.8)
≥ 25	52 (45.2)
Hypertension, n (%)	
No	96 (83.5)
Yes	19 (16.5)
Diabetes, n (%)	
No	95 (82.6)
Yes	20 (17.4)
Smoking, n (%)	
No	93 (80.9)
Yes	22 (19.1)
Alcohol abuse, n (%)	
No	99 (86.1)
Yes	16 (13.9)
Hemoglobin (g/L), median (IQR)	131 (122–140)
Prealbumin (g/L), median (IQR)	210 (184–251)
Albumin (g/L), n (%)	
≥ 35	90 (78.3)
< 35	25 (21.7)
CA199 (KU/L), median (IQR)	16.2 (5.80–76.8)
Operation time (min), median (IQR)	287 (231–352)
Blood loss (mL), median (IQR)	300 (200–550)
Surgical approach, n (%)	
Laparoscopic	16 (13.9)
Open	99 (86.1)
Ligation of main pancreatic duct, n (%)	
No	54 (47.0)
Yes	61 (53.0)
Pancreatic stump treatment, n (%)	
Endo GIA stapler	30 (26.1)
Suture	85 (73.9)
Pathology, n (%)	
PDAC	36 (31.3)
Cystic	45 (39.13)
Pancreatitis	10 (8.7)
Neuroendocrine	13 (11.3)
SPTP	9 (7.83)
Others	2 (1.74)
Pancreas thickness (mm), median (IQR)	17.2 (15.3–20.2)
Pancreas texture, n (%)	
Hard	69 (60.0)
Soft	46 (40.0)
Splenectomy, n (%)	
No	56 (48.7)
Yes	59 (51.3)
CR-POPF, n (%)	
No	77 (67.0)
Yes	38 (33.0)

*BMI* body mass index; *IQR* interquartile range; *CA199* cancerantigen199; *PDAC* pancreatic ductal adenocarcinoma; *SPTP* solid pseudopapillary tumor of the pancreas

### Statistical analysis

Continuous variables were expressed as the means and standard deviations or medians and interquartile ranges (IQR) and compared by the Mann–Whitney U test, as appropriate. Categorical variables are presented as the counts and percentages in each category and were compared by the chi-squared test or Fisher’s exact test. All variables associated with CR-POPF at a significant level were candidates for stepwise multivariate analysis. A nomogram was formulated based on the results of multivariate logistic regression analysis and by using the vrpm package of R version 4.1.3 (http://mirror.bjtu.edu.cn/cran/bin/windows/base/). The predictive performance of the nomogram was measured by the concordance index (C index) and calibration with 1000 bootstrap samples to decrease the overfit bias. The clinical application value of this model was validated using decision curve and clinical impact curve analyses. All statistical analyses were performed using R software studio (version 4.1.3), and a P value of less than 0.05 was considered to be statistically significant [37].

## Results

### Clinicopathologic characteristics of the study cohort

In total, 115 patients were included in the present study, of which 34 were men and 81 women, with a median age of 53.0 years (45.5–62.0). Of these 115 patients, 25 (21.7%) had preoperative hypoalbuminemia, and 46 (40.0%) had a soft pancreas. The median pancreas thickness was 17.2 mm. Among these patients, 99 underwent open surgery, and 16 underwent laparoscopic surgery. The median operative time was 287 min, and the median blood loss was 300 ml. There were 59 patients who underwent combined splenectomy, whereas the spleen was preserved in 56 patients. During the postoperative follow-up and management, 77 (67.0%) patients did not present CR-POPF, while 38 (33.0%) developed CR-POPF. The clinicopathologic characteristics are presented in Table 1.

### Independent risk factors associated with CR-POPF

All 115 patients were divided into the CR-POPF group (n = 38) and the non-CR-POPF group (n = 77) based on the diagnosis of CR-POPF. The results of univariate analysis showed that patients with higher BMI (P = 0.004), hypertension history (P = 0.024), lower serum prealbumin level (P = 0.032), lower serum albumin level (P = 0.001), a thicker pancreas (P < 0.001) and a soft pancreas (P = 0.001) were more likely to develop CR-POPF (Table 2). All of the abovementioned significant parameters were then put into multivariate logistic regression analysis. The results showed that higher BMI (OR 4.658, 95% CI 1.716–14.10, P = 0.004), lower serum albumin level (OR 7.934, 95% CI 2.548–28.292, P = 0.001), thicker pancreas (OR 1.256, 95% CI 1.086–1.470, P = 0.003) and soft pancreas (OR 3.143, 95% CI 1.203–8.497, P = 0.021) were independent risk factors associated with CR-POPF following DP (Table 3).

**Table 2:** Univariate analysis of risk factors for postoperative pancreatic fistula after DP

Variable	No CR-POPF (N = 77)	CR-POPF (N = 38)	*P* value
Gender, n (%)			0.908
Male	22 (28.6)	12 (31.6)	
Female	55 (71.4)	26 (68.4)	
Age (y), n (%)			0.514
< 65	66 (85.7)	30 (78.9)	
≥ 65	11 (14.3)	8 (21.1)	
BMI (kg/m^2^), n (%)			0.004
< 25	50 (64.9)	13 (34.2)	
≥ 25	27 (35.1)	25 (65.8)	
Hypertension, n (%)			0.024
No	69 (89.6)	27 (71.1)	
Yes	8 (10.4)	11 (28.9)	
Diabetes, n (%)			0.130
No	67 (87.0)	28 (73.7)	
Yes	10 (13.0)	10 (26.3)	
Smoking, n (%)			0.908
No	63 (81.8)	30 (78.9)	
Yes	14 (18.2)	8 (21.1)	
Alcohol abuse, n (%)			0.487
No	68 (88.3)	31 (81.6)	
Yes	9 (11.7)	7 (18.4)	
Hemoglobin (g/L), median (IQR)	130 (122–140)	131 (124–138)	0.917
Prealbumin (mg/L), median (IQR)	210 (191–253)	192 (175–240)	0.032
Albumin (g/L), n (%)			0.001
≥ 35	68 (88.3)	22 (57.9)	
< 35	9 (11.7)	16 (42.1)	
CA199 (kU/L), median (IQR)	14.6 (5.1–36.0)	28.9 (13.6–296)	0.061
Operation time (min), median (IQR)	285 (240–345)	314 (214–364)	0.861
Blood loss (mL), median (IQR)	300 (200–500)	320 (200–600)	0.691
Surgical approach, n (%)			0.903
Laparoscopic	10 (13.0)	6 (15.8)	
Open	67 (87.0)	32 (84.2)	
Ligation of main pancreatic duct, n (%)			0.794
Yes	35 (45.5)	19 (50.0)	
No	42 (54.5)	19 (50.0)	
Pancreatic stump treatment, n (%)			0.791
Endo GIA stapler	19 (24.7)	11 (28.9)	
Suture	58 (75.3)	27 (71.1)	
Pathology, n (%)			0.715
PDAC	22 (28.6)	14 (36.8)	
Cystic	30 (39.0)	15 (39.5)	
Pancreatitis	7 (9.1)	3 (7.9)	
Neuroendocrine	11 (14.3)	2 (5.3)	
SPTP	6 (7.8)	3 (7.9)	
Others	1 (1.2)	1 (2.6)	
Pancreas thickness (mm), median (IQR)	16.4 (14.9–18.6)	19.5 (16.8–22.5)	< 0.001
Pancreas texture, n (%)			0.001
Hard	55 (71.4)	14 (36.8)	
Soft	22 (28.6)	24 (63.2)	
Splenectomy, n (%)			0.693
No	36 (46.8)	20 (52.6)	
Yes	41 (53.2)	18 (47.4)	

*BMI* body mass index; *IQR* interquartile range; *CA199* cancerantigen199; *PDAC* pancreatic ductal adenocarcinoma; *SPTP* solid pseudopapillary tumor of the pancreas

**Table 3:** Multivariate logistic regression analysis for postoperative pancreatic fistula after DP

Variable	β	S.E.	Wald	*P* value	OR	95% CI
BMI, kg/m^2^, (≥ 25 vs < 25)	1.539	0.531	2.898	0.004	4.658	1.716–14.10
Albumin, g/L, (< 35 vs ≥ 35)	2.071	0.607	3.413	0.001	7.934	2.548–28.292
Pancreas thickness, mm	0.228	0.076	2.984	0.003	1.256	1.086–1.470
Pancreas texture, (soft vs hard)	1.145	0.495	2.314	0.021	3.143	1.203–8.497

*BMI* body mass index; *β* regression coefficient; *S.E.* standard error of regression coefficient; *Wald* Wald chi-square value; *CI* confidence interval; *OR* odds ratio

### Construction of a predictive nomogram incorporating risk factors for CR-POPF

**Fig. 1:**
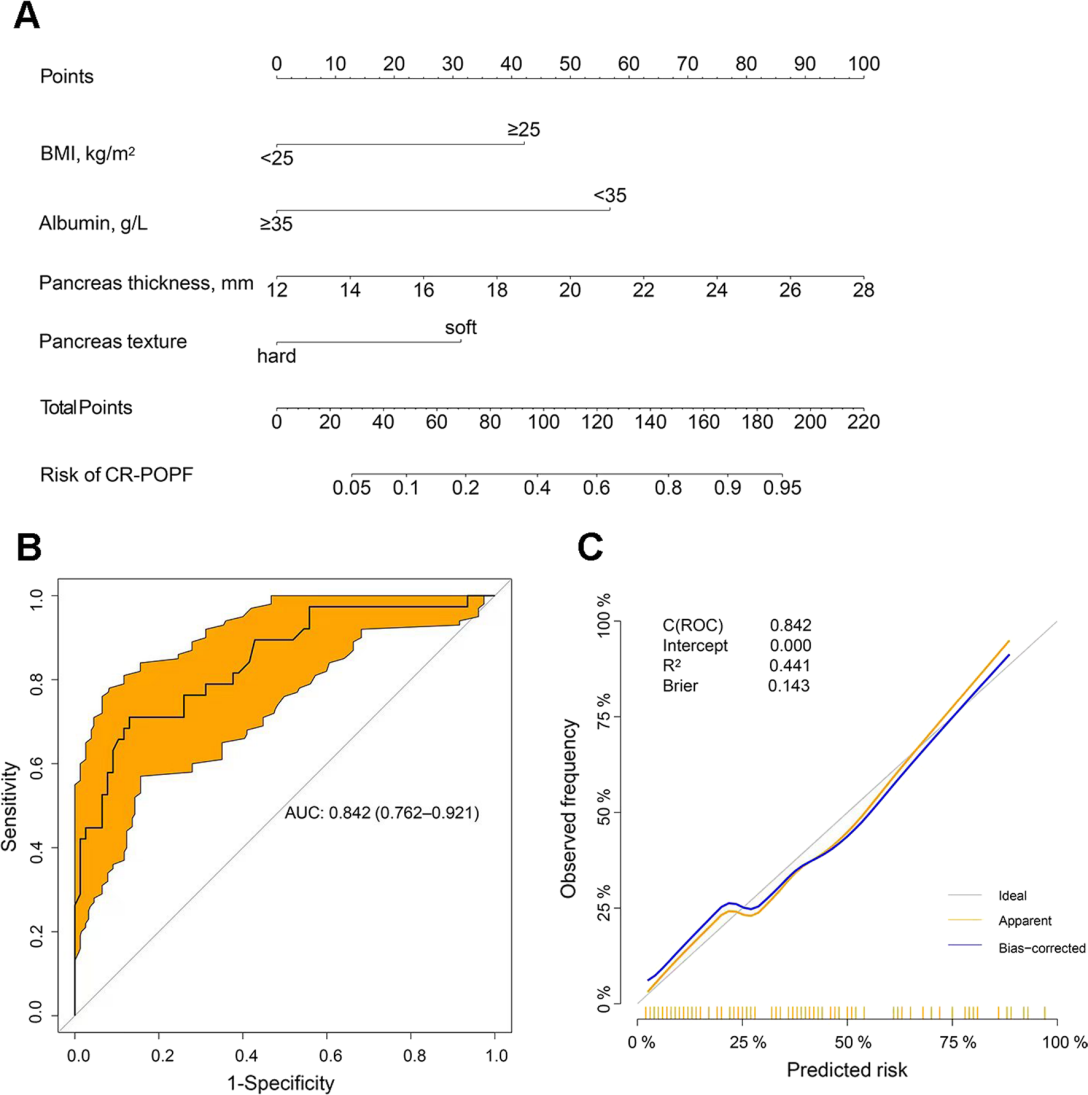
**A** Nomogram for preoperative prediction of CR-POPF following DP. Points indicate BMI, preoperative serum albumin level, pancreatic thickness and pancreatic texture. The score for each value was assigned by drawing a line upwards to the “Points” line, and the sum of the four scores was plotted on the “Total points” line (probability of CR-POPF). **B** Receiver operating characteristic (ROC) curves were used to evaluate the nomogram model performance. **C** The calibration curve of the nomogram model

**Fig. 2:**
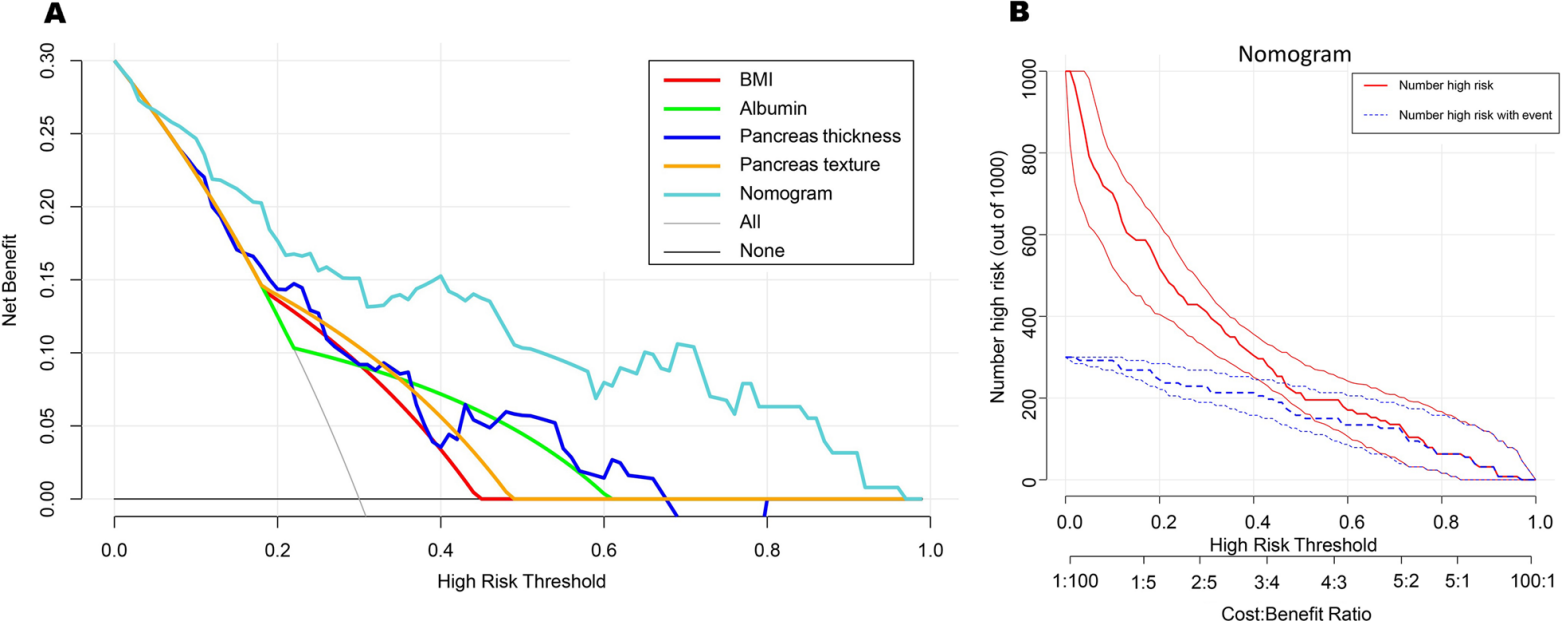
**A** Decision curve analysis of the nomogram model. **B** A clinical impact curve of the nomogram model

The independent risk factors associated with CR-POPF were used to construct a nomogram (Fig. 1A). The nomogram demonstrated good accuracy in estimating the risk of CR-POPF, with a C-index of 0.842 (95% CI 0.762–0.921) (Fig. 1B). Calibration plots graphically exhibited good consistency between actual observations and nomogram-predicted CR-POPF (Fig. 1C). The predictive value of the nomogram, including AUC, sensitivity, specificity, positive predictive value and negative predictive value, was compared with each risk factor in the present study. The optimal cut-off value of total nomogram scores was determined to be 102. The results showed that the C-index of the nomogram was 0.842 (95% CI 0.762–0.921), which was significantly higher than that of each indictor alone [BMI: 0.654 (95% CI 0.560–0.747), albumin: 0.652 (95% CI 0.565–0.739), pancreas thickness: 0.722 (95% CI 0.620–0.824), pancreas texture: 0.673 (95% CI 0.580–0.766)]. Compared to each indictor, the nomogram predicted CR-POPF with a sensitivity of 0.710 and specificity of 0.870, yielding a PPV of 0.730 and NPV of 0.859, indicating that the nomogram had better discriminatory performance (Table 4). Moreover, decision curve analysis (DCA) and clinical impact curve (CIC) were used to validate the clinical application value of the model. As shown in Fig. 2A, B, the nomogram also showed greater clinical net benefits, which further demonstrated that the nomogram had better predictive and accuracy values.

**Table 4:** Discriminatory performance of BMI, albumin, indication of surgery, pancreas thickness, pancreas texture, and the formulated nomogram for detecting patients with CR-POPF after PD

Variables	AUC (95% CI)	Cut off	Sensitivity	Specificity	PPV	NPV
BMI	0.654 (0.560–0.747)	–	0.658	0.649	0.481	0.794
Albumin	0.652 (0.565–0.739)	–	0.421	0.883	0.640	0.756
Pancreas thickness	0.722 (0.620–0.824)	19.3	0.579	0.779	0.564	0.789
Pancreas texture	0.673 (0.580–0.766)	–	0.632	0.714	0.522	0.797
Nomogram	0.842 (0.762–0.921)	102	0.710	0.870	0.730	0.859

*BMI* body mass index; *AUC* area under the receiver-operating-characteristic curve; *CI* confidence interval; *PPV* positive predictive value; *NPV* negative predictive value

## Discussion

CR-POPF following DP has been considered a potential precursor of more serious events. In the present study, the incidence of CR-POPF was 33.0%, which is similar to the results of previous studies [4, 10–18]. Our study also suggested that BMI, preoperative serum albumin level, pancreatic thickness and pancreatic texture are significantly associated with CR-POPF after DP.

Previous studies [38–40] have attempted to use drain fluid amylase levels on the first postoperative day to predict CR-POPF following DP, but further clinical validation is needed. There is a wide range in cut-off values between these studies, which would limit its wide use. One study [41] reported a postoperative score that incorporated four factors (i.e., operation time, BMI, amylase level on drains on postoperative Day 3 and pancreatic thickness) to predict the risk of developing CR-POPF. However, other risk factors that have been recognized as important for CR-POPF development were not included in the model. Nomograms, an easy-to-use prediction tool, have been widely used to predict clinical events. In the present study, we constructed a nomogram by incorporating four comprehensive and easily available variables. Importantly, this nomogram showed satisfactory discriminative ability and accuracy.

All four risk factors used to construct this nomogram have been reported in previous studies. A higher BMI is generally accepted as an important risk factor for the development of POPF following DP [18, 42]. It is well known that a higher BMI increases intraoperative technical difficulty and influences the physiology of the pancreas because of pancreatic fatty infiltration [43, 44]. Increased fat in the pancreas would intuitively increase the softness of the gland. Indeed, the soft pancreatic texture is known to be an important risk factor for fistula development following pancreatomy [17, 18]. Although there are no standardized criteria to define the texture of the pancreas, pancreatic texture has already been used to evaluate the risk of POPF after pancreatic resection, especially PD. Intraoperative ultrasound elastography may be useful for determining the pancreatic texture [45], but it is not routinely used in the operation, and its diagnostic performance needs to be further validated. In the present study, as a conventional approach, the pancreatic texture was determined by two experienced surgeons during the operation. Moreover, there is compelling evidence proving that the POPF rate increases as thickness increases [18, 36, 46]. Patients with a thicker pancreas would increase the technical difficulties of suturing or stapling, which may be why recent technological innovations do not significantly decrease the rate of CR-POPF after DP. Preoperative serum albumin levels have also been demonstrated to be a predictive factor for CR-POPF after DP [18]. Preoperative hypoalbuminemia is often correlated with increased morbidity after surgery, as reported in many studies [47, 48]. The explanations of this result include poor tissue healing, decreased collagen synthesis in surgical wounds, delayed return of bowel function and suppression of the systemic inflammatory response. Unsurprisingly, the proposed nomogram, which incorporated the abovementioned variables, performed well, as supported by the C index values of 0.842, and showed better discriminatory performance to determine CR-POPF, with a sensitivity of 0.710, specificity of 0.870, PPV of 0.730 and NPV of 0.859 when compared to each variable. Furthermore, the nomogram also presented a high clinical net benefit in predicting CR-POPF.

All variables used in this nomogram are universal and easily available. Based on the predictive accuracy of CR-POPF, we believe this nomogram model would help surgeons make reasonable decisions to prevent the occurrence of severe adverse events. First, patients with albumin < 35 g/L should have nutrition supplementation prior to surgery. Second, it is difficult to close the stump of the remnant pancreas completely when patients have a soft pancreas or a thick pancreas. A pancreatic duct stent was placed prior to surgery, and a combination of linear stapling plus continuous suturing of the stump would be optimal to decrease the incidence of CR-POPF. Third, this nomogram model could accurately stratify patients with different risks of CR-POPF, which enables surgeons to choose a reasonable drainage strategy. For patients with a low risk of CR-POPF, early drain removal should be performed safely, whereas patients with total scores greater than 102 should receive more attention and take more effective measures.

This study has some limitations that should be mentioned. First, this was a single-centre retrospective study with a limited sample size. Moreover, this model was not externally validated. Further validation needs to be performed in other institutions with large sample sizes. Second, other factors, which might be correlated with postoperative CR-POPF, such as drain fluid amylase level, were not included in this study. Finally, although the nomogram exhibited good predictive accuracy, the false-positive rate was 0.270, and the false-negative rate was 0.141 for predicting CR-POPF presence, which remains high if major clinical decisions are needed.

## Conclusion

We identified BMI, preoperative serum albumin level, pancreatic thickness and pancreatic texture as the preoperative factors for CR-POPF following DP. By combining these preoperative factors, a nomogram was constructed. The nomogram provides an optimal preoperative estimation of CR-POPF presence in patients who undergo DP.

## Data Availability

The data that support the findings of this study are available on request from the corresponding author. The data are not publicly available due to privacy or ethical restrictions.

## Abbreviations

POPF: Postoperative pancreatic fistula
DP: Distal pancreatectomy
CR-POPF: Clinically relevant postoperative pancreatic fistula
ROC: Receiver operating characteristic
BMI: Body mass index
FRS: Fistula risk score
NGT: Nasogastric tube
CT: Computed tomography
IQR: Interquartile ranges
DCA: Decision curve analysis
CIC: Clinical impact curve
